# Adrenal gland involvement in 11-ketotestosterone production analyzed using LC-MS/MS

**DOI:** 10.3389/fendo.2023.1051195

**Published:** 2023-01-20

**Authors:** Kento Ikegawa, Yukihiro Hasegawa

**Affiliations:** ^1^ Division of Endocrinology and Metabolism, Tokyo Metropolitan Children’s Medical Center, Tokyo, Japan; ^2^ Clinical Research Support Center, Tokyo Metropolitan Children’s Medical Center, Tokyo, Japan; ^3^ Department of Pediatrics, Keio University of School of Medicine, Tokyo, Japan

**Keywords:** 11-ketotestosterone, 11-hydroxytestosterone, 11-ketodihydrotestosterone, LC-MS/MS, adrenal grands, androgen

## Abstract

**Introduction:**

11-ketotestosterone (11KT), which is derived by the bioconversion of testosterone *via* 11β-hydroxytestosterone (11OHT), is a potent agonist of the human androgen receptor. The adrenal gland is considered an important organ in 11KT production because CYP11B1, which catalyzes testosterone to 11OHT, is expressed in the adrenal glands. The present study aimed to demonstrate adrenal gland involvement in 11KT production in prepubertal children, a topic which has not yet been addressed by any previous studies.

**Methods:**

Three, retrospective, observational studies were performed. Study 1 enrolled patients aged 8 months to 7 years with severe Kawasaki disease (KD) who were treated with mPSL pulse. Studies 2 and 3 included patients who had received a corticotropin-releasing hormone (CRH) stimulation test and adrenocorticotropic hormone (ACTH) stimulation test, respectively. Samples were collected before and after treatment or drug administration, and serum 11KT, 11OHT, and other 11-oxygenated androgens were measured by LC-MS/MS. Steroid hormone values before and after medication were analyzed using the Wilcoxon signed rank test.

**Results:**

Studies 1, 2, and 3 included twenty patients with severe KD, eight patients with a CRH stimulation test, and eight patients with an ACTH stimulation test, respectively. Study 1 demonstrated that the median (IQR) 11KT level was significantly higher before, than after, mPSL pulse (0.39 (0.28-0.47) nmol/L versus 0.064 (0.012-0.075) nmol/L; P < 0.001). Studies 2 and 3 indicated no significant difference in the median 11KT value before and after the CRH or ACTH stimulation test while the 11OHT value was significantly higher after the test.

**Conclusion:**

In conclusion, the mediation of 11KT production by ACTH demonstrated the importance of the adrenal glands in the synthesis of this androgen in prepubertal children.

## 1 Introduction

Testosterone and dihydrotestosterone are major ligands of androgen receptors (1). In recent years, 11-ketotestosterone (11KT) and 11-ketodihydrotestosterone (11KDHT), which belong to the group of 11-oxygenated androgens, have been attracting attention as potent agonists of the human androgen receptor because they have activity similar to that of testosterone (2–4). 11KT in particular is thought to play an important role in several diseases characterized by androgen excess, such as congenital adrenal hyperplasia, premature adrenarche, and polycystic ovary syndrome (5–7). Previous reports demonstrated that 11KT was 3.4 times higher in patients with classic 21-hydroxylase deficiency (21OHD) than in control subjects (8) and was also higher in pre-adult female subjects with premature adrenarche than in their age-matched, normal counterparts (9).

**Figure 1:**
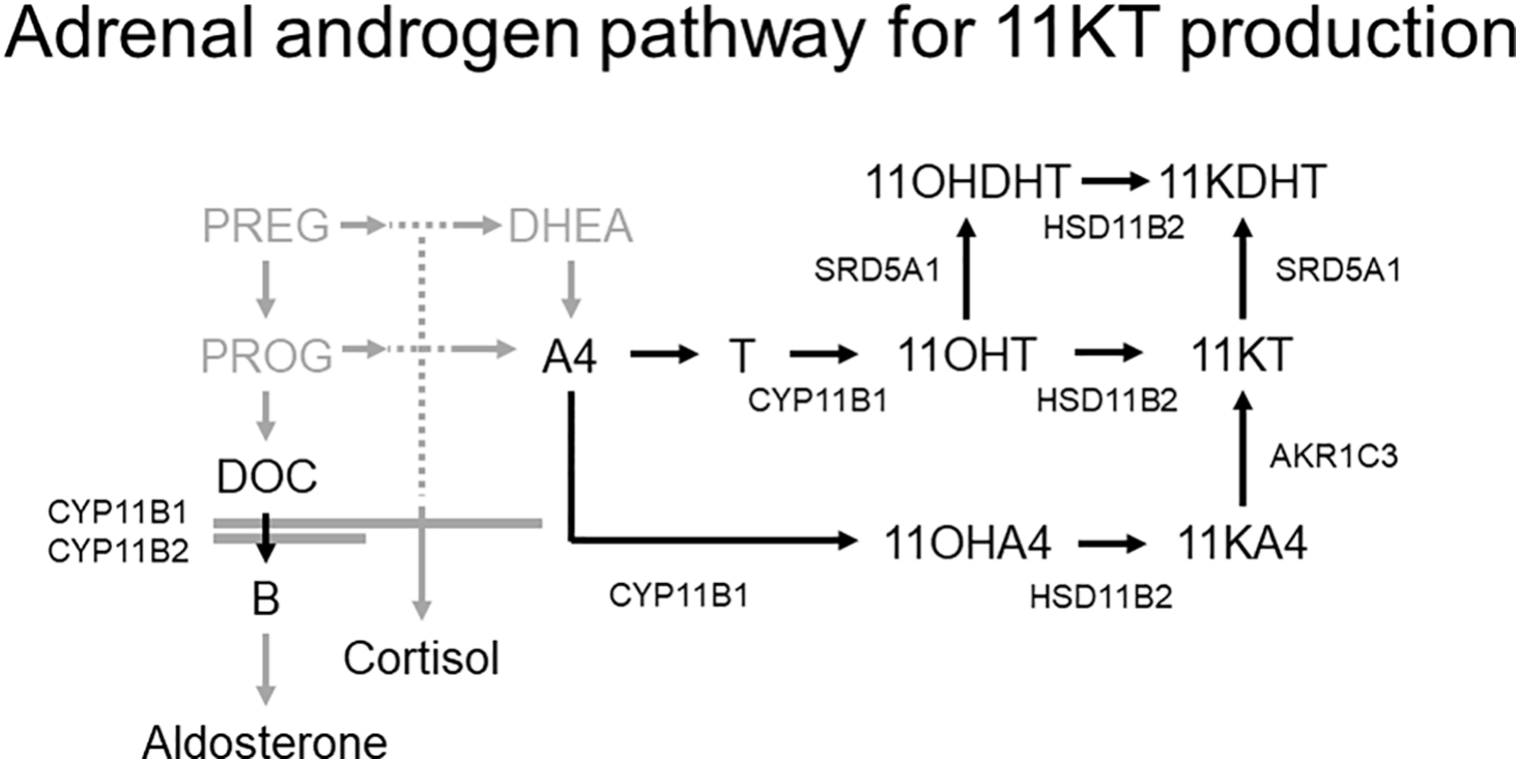
Adrenal androgen pathway for 11KT production (6). Two pathways produce 11KT; one involves the conversion of testosterone to 11OHT, and the other involves the conversion of A4 to 11KA4 *via* 11OHA4. The conversion of testosterone and A4 to 11OHT and 11OHA4, respectively, is catalyzed by CYP11B1, and the subsequent conversion of 11OHT and 11OHA4 to 11KT and 11KA4, respectively, is catalyzed by HSD11B2.

Two pathways produce 11KT. One of these converts testosterone into 11KT *via* 11β-hydroxytestosterone (11OHT) while the other converts androstenedione (A4) *via* 11-ketoandrostenedione (11KA4) derived from 11β-hydroxyandrostenedione (11OHA4) (**Figure 1**) (6). In the first pathway, the first reaction, in which testosterone is converted to 11OHT, is catalyzed by cytochrome P450 11β-hydroxylase (CYP11B1); the second reaction, in which 11OHT is converted to 11KT, is catalyzed by 11β-hydroxysteroid dehydrogenase type 2 (HSD11B2) (6). CYP11B1, which also catalyzes the conversion of deoxycorticosterone (DOC) and 11-deoxycortisol (11DOF) to corticosterone (B) and cortisol, respectively, under the regulation of adrenocorticotropic hormone (ACTH), is expressed in the zonae fasciculata and reticularis of the adrenal glands (10). HSD11B2, which also converts cortisol to cortisone, is widely expressed in various organs, including the kidneys, colon, sweat glands, salivary glands, and placenta (11, 12). In the latter pathway, the first reaction, converting A4 to 11OHA4, and the second reaction, converting 11OHA4 to 11KA, are catalyzed by CYP11B1 and HSD11B2, respectively, the same enzymes as in the first pathway. The final reaction in the second pathway, in which 11KA is converted to 11KT, is catalyzed by aldo-keto reductase family 1 member C3 (AKR1C3), which is expressed in various human tissues, including the adrenal glands (6).

The adrenal gland is considered an important organ in the production of 11KT because it expresses CYP11B1 (5, 10). A previous study demonstrated that the size of the adrenal glands in patients with 21OHD correlated with their 11KT level, suggesting adrenal gland involvement in 11KT production (7). Some studies using a murine model suggested that the gonads also synthesize 11KT (2, 13), but as far as we know, there are no such findings in humans. Thus, while previous reports indicated that the adrenal glands play an important role in 11KT production, there are only a few reports of children without gonadotropin releasing hormone (GnRH) activation. Moreover, no studies have as of yet examined 11KT during suppression of adrenal function or compared 11KT before and after adrenal stimulation.

The present study aimed to demonstrate adrenal gland involvement in 11KT production in prepubertal children without GnRH activation, a topic which has not been addressed in previous studies.

## 2 Method

### 2.1 Study design and participants

Three, retrospective, observational studies including prepubertal children whose samples were stored were performed at Tokyo Metropolitan Children’s Medical Center in Tokyo, Japan. Study 1 enrolled patients aged between 8 months and 7 years with severe Kawasaki disease (KD) who were treated between April 2017 and March 2019. Severe KD was defined as a Kobayashi risk score ≥5 (14). All patients with severe KD admitted to our hospital receive intravenous immunoglobulin 2 g/kg, methylprednisolone (mPSL) pulse 30 mg/kg, and oral aspirin 30 mg/kg/day, followed by oral prednisolone 2 mg/kg/day and oral aspirin 5 mg/kg/day. Study 2 included patients of an age similar to that of the patients in study 1 who received a corticotropin-releasing hormone (CRH) stimulation test using intravenous CRH 1.0 µg/kg between January and July 2020. Study 3 included patients aged <10 years who received an ACTH stimulation test using intravenous ACTH 0.25 mg/m^2^ or 1.0 µg/kg between September 2020 and July 2021.

### 2.2 Timing of the blood testing and method of serum steroid analysis

Samples collected before, and five to nine days after, the treatment in study 1 and before, and at 60 minutes after, drug administration in studies 2 and 3 were used for testing. Serum cortisol, 11KT, 11OHT, and 11KDHT were measured at each time point using LC-MS/MS (ASKA Pharma Medical Co., Ltd., Kanagawa, Japan) (15). Only the pathway of 11KT production from testosterone *via* 11OHT was investigated because 11KT is produced by similar enzymes in both pathways, suggesting the possibility of similar results.

### 2.3 Ethical considerations

The present study was approved by the ethics committee at Tokyo Metropolitan Children’s Medical Center and was performed in accordance with the ethical standards laid down in the Declaration of Helsinki and the Strengthening the Reporting of Observational studies in Epidemiology (STROBE) guidelines. Because no additional blood tests were performed, and stored samples were used, the ethics committee waived the requirement for written informed consent.

### 2.4 Statistical analysis

The patient characteristics were described in terms of the median and interquartile range (IQR) for continuous variables and the frequency and proportion for categorical variables. Steroid hormone values before and after medication were analyzed using the Wilcoxon signed rank test. Pearson’s correlation coefficient was used to assess for any association between 11KT and 11OHT. The *P* values were 2-tailed with P < 0.05 indicating statistical significance. All statistical analyses were performed with SPSS version 27 (IBM Corp., Armonk, NY, US).

## 3 Results

Twenty patients aged between 8 months and 7 years with severe KD who received mPSL pulse 30 mg/kg, eight patients with a CRH stimulation test, and eight patients with an ACTH stimulation test were included in study 1, 2, and 3, respectively. The median (IQR) values for age were 2.9 (2.0-3.7) years, 3.8 (2.0-4.1) years, and 4.2 (0.2-7.3) years for the respective studies (**Table 1**).

**Table 1:** Patient characteristics.

	Study 1, KD patients (n = 20)	Study 2, CRH stimulation test (n = 8)	Study 3, ACTH stimulation test (n = 8)
Age (y), median, IQR	2.9 (2.0-3.7)	3.8 (2.0-4.1)	4.2 (0.2-7.3)
Male sex, n (%)	9 (45)	5 (62.5)	3 (37.5)
HDC equivalent dose (mg/m2), median, IQR	4362.7 (4189.4-4826.7)	–	–
Duration of steroid treatment (d), median, IQR	7.5 (7.0-8.8)	–	–
F level^†^ before medication (nmol/L), median, IQR	1051.2 (739.4-1209.8)	171.1 (113.8-209.7)	166.9 (163.5-223.5)
F level^†^ after medication (nmol/L), median, IQR	67.0 (53.2-123.6)	478.7 (366.9-627.7)	533.9 (340.0-855.3)

KD, Kawasaki disease; CRH, corticotropin-releasing hormone; ACTH, adrenocorticotropic hormone; F, cortisol; IQR, interquartile range.

^†^Serum cortisol was measured using ECLIA.

**Figure 2:**
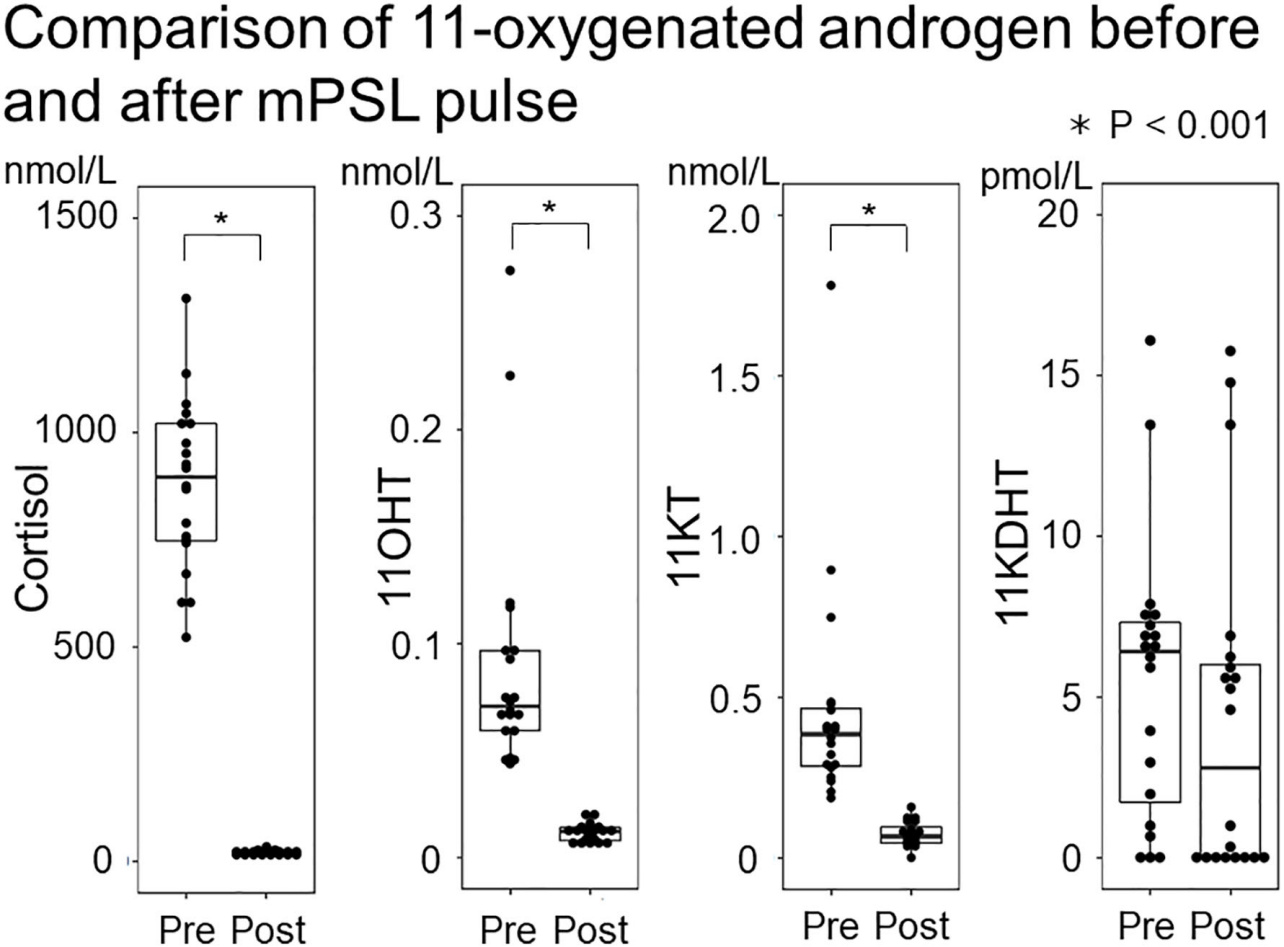
Comparison of 11-oxygenated androgen before and after mPSL pulse. The 11-oxygenated androgen values before and after mPSL pulse were compared. Serum cortisol, 11OHT, and 11KT were significantly lower after mPSL pulse.

**Figure 3:**
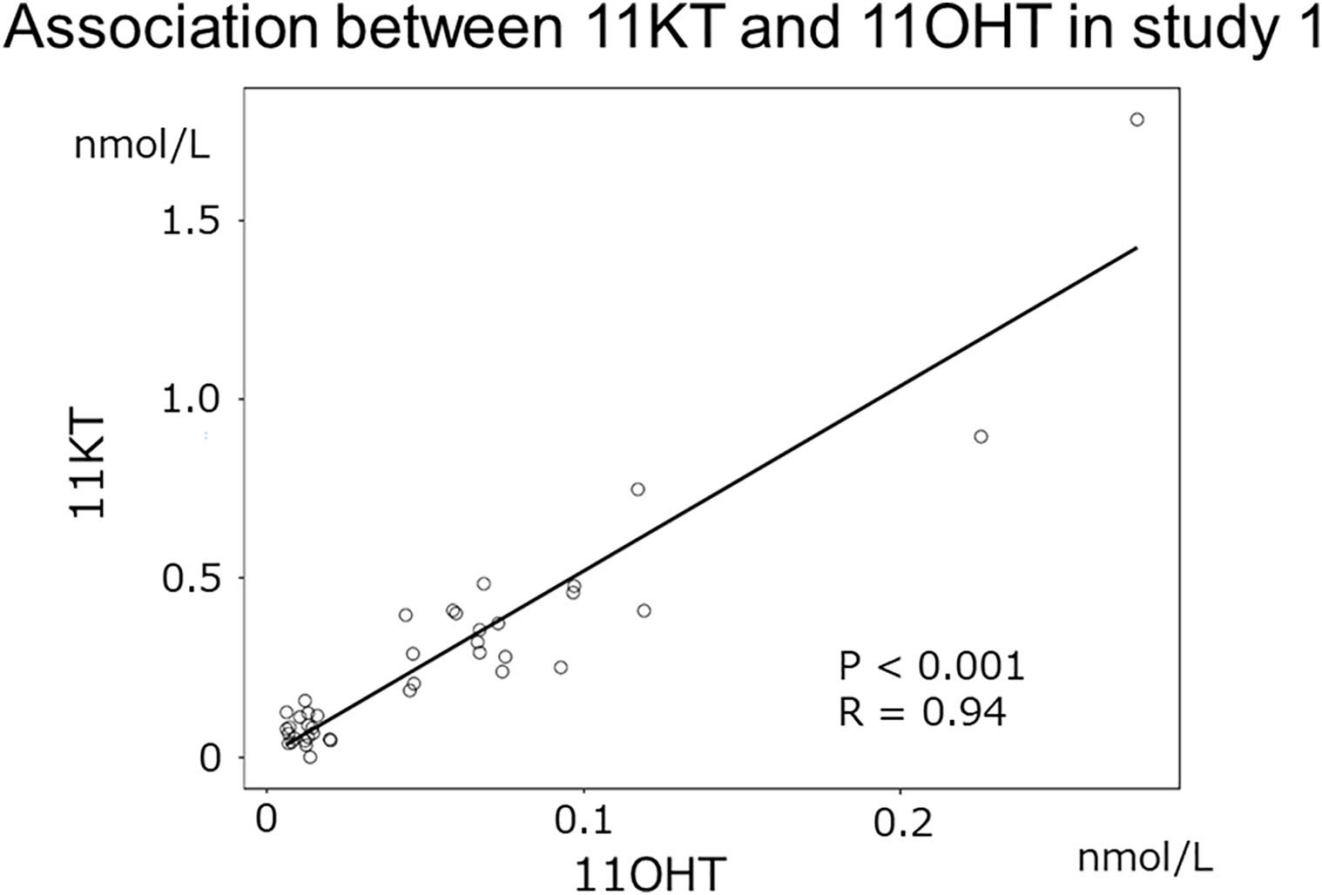
Association between 11KT and 11OHT in study 1. The scatter plot shows the association between 11KT and 11OHT in study 1 (Pearson’s correlation coefficient: 0.94 [P < 0.001]).

Study 1 demonstrated that the median (IQR) of 11KT was significantly higher before mPSL pulse than after mPSL pulse at 0.39 (0.28-0.47) nmol/L and 0.064 (0.012-0.075) nmol/L, respectively (**Table 2**). The 11OHT value was also significantly higher before, than after, mPSL pulse (**Figure 2**). Pearson’s correlation coefficient between 11KT and 11OHT was 0.94 (P < 0.001) (**Figure 3**). No sex difference was observed for any of the steroid hormone values (**Table 3**).

**Table 2:** Steroid hormone values before and after mPSL pulse (Study 1).

	Before mPSL pulse	After mPSL pulse	P value^†^
F (nmol/L), median, IQR	896.2 (744.7-1021.8)	22.3 (15.3-24.3)	< 0.001^‡^
11KT (nmol/L), median, IQR	0.39 (0.28-0.47)	0.064 (0.012-0.075)	< 0.001^‡^
11OHT (nmol/L), median, IQR	0.071 (0.059-0.097)	0.012 (0.0073-0.014)	< 0.001^‡^
11KDHT (pmol/L), median, IQR	6.4 (1.2-7.5)	2.8 (0-6.2)	0.3^‡^

mPSL, methylprednisolone; F, cortisol; 11KT, 11-ketotestosterone; 11OHT, 11-hydroxytestosterone; 11KDHT, 11-ketodihydrotestosterone; IQR, interquartile range.

^†^Univariate analysis was performed, and P <.05 was considered to indicate statistical significance.

^‡^Wilcoxon signed-rank test.

**Table 3:** Sex difference in steroid hormone values (Study 1).

	Before mPSL pulse		After mPSL pulse	
	Males (n = 9)	Females (n = 11)	Males (n = 9)	Females (n = 11)
F (nmol/L), median, IQR	917.0 (773.4-1056.3)	868.1 (604.4-1022.5)	23.5 (16.8-25.7)	17.4 (15.0-22.9)
11KT (nmol/L), median, IQR	0.32 (0.22-0.82)	0.40 (0.29-0.41)	0.052 (0.042-0.074)	0.083 (0.047-0.12)
11OHT (nmol/L), median, IQR	0.093 (0.056-0.17)	0.067 (0.059-0.075)	0.012 (0.0078-0.014)	0.013 (0.0071-0.015)
11KDHT (pmol/L), median, IQR	7.2 (3.4-10.5)	5.9 (0-6.9)	4.6 (0-5.9)	0.33 (0-6.8)

mPSL, methylprednisolone; F, cortisol; 11KT, 11-ketotestosterone; 11OHT, 11-hydroxytestosterone; 11KDHT, 11-ketodihydrotestosterone; IQR, interquartile range.

**Figure 4:**
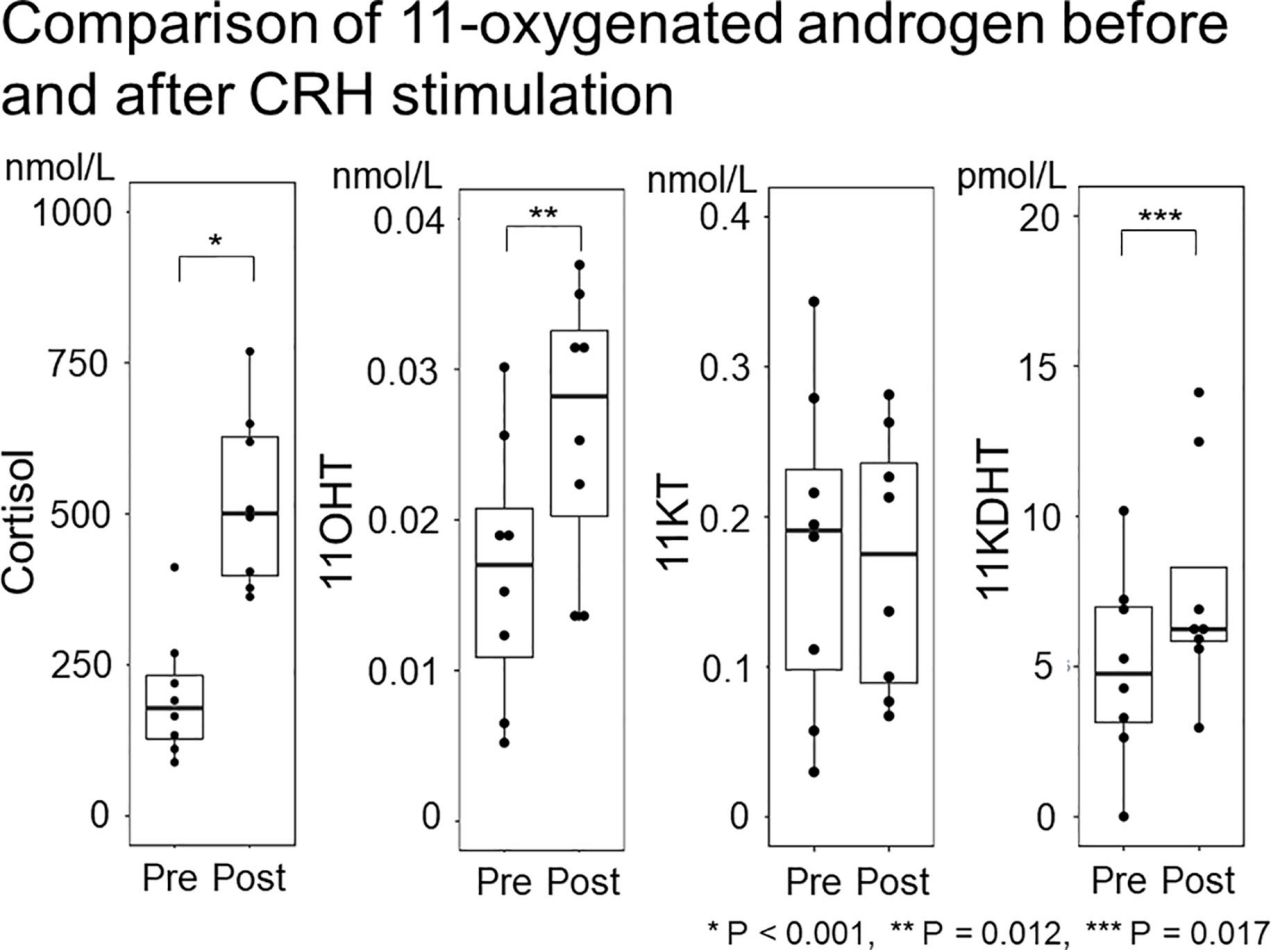
Comparison of 11-oxygenated androgen before and after CRH stimulation. The 11-oxygenated androgen values before and after CRH stimulation were compared. Serum cortisol and 11OHT were higher after CRH stimulation, but 11KT did not differ before or after ACTH stimulation.

Study 2 demonstrated no significant difference in the median (IQR) 11KT value before and after the CRH stimulation test (0.19 (0.071-0.26) nmol/L and 0.17 (0.081-0.25) nmol/L; P = 0.889) while the 11OHT and 11KDHT values were significantly higher after the CRH stimulation test than before it (**Table 4**; **Figure 4**).

**Table 4:** Steroid hormone values before and at 60 minutes after CRH administration (Study 2).

	Before CRH administration	60 minutes after CRH administration	P value^†^
F (nmol/L), median, IQR	177.7 (116.1-256.5)	500.9 (384.0-641.7)	< 0.001^‡^
11KT (nmol/L), median, IQR	0.19 (0.071-0.26)	0.17 (0.081-0.25)	0.889^‡^
11OHT (nmol/L), median, IQR	0.017 (0.0079-0.024)	0.028 (0.016-0.034)	0.012^‡^
11KDHT (pmol/L), median, IQR	4.8 (2.8-7.1)	6.2 (5.7-11.1)	0.017^‡^

CRH, corticotropin-releasing hormone; F, cortisol; 11KT, 11-ketotestosterone; 11OHT, 11-hydroxytestosterone; 11KDHT, 11-ketodihydrotestosterone; IQR, interquartile range.

^†^Univariate analysis was performed, and P <.05 was considered to indicate statistical significance.

^‡^Wilcoxon signed-rank test.

**Figure 5:**
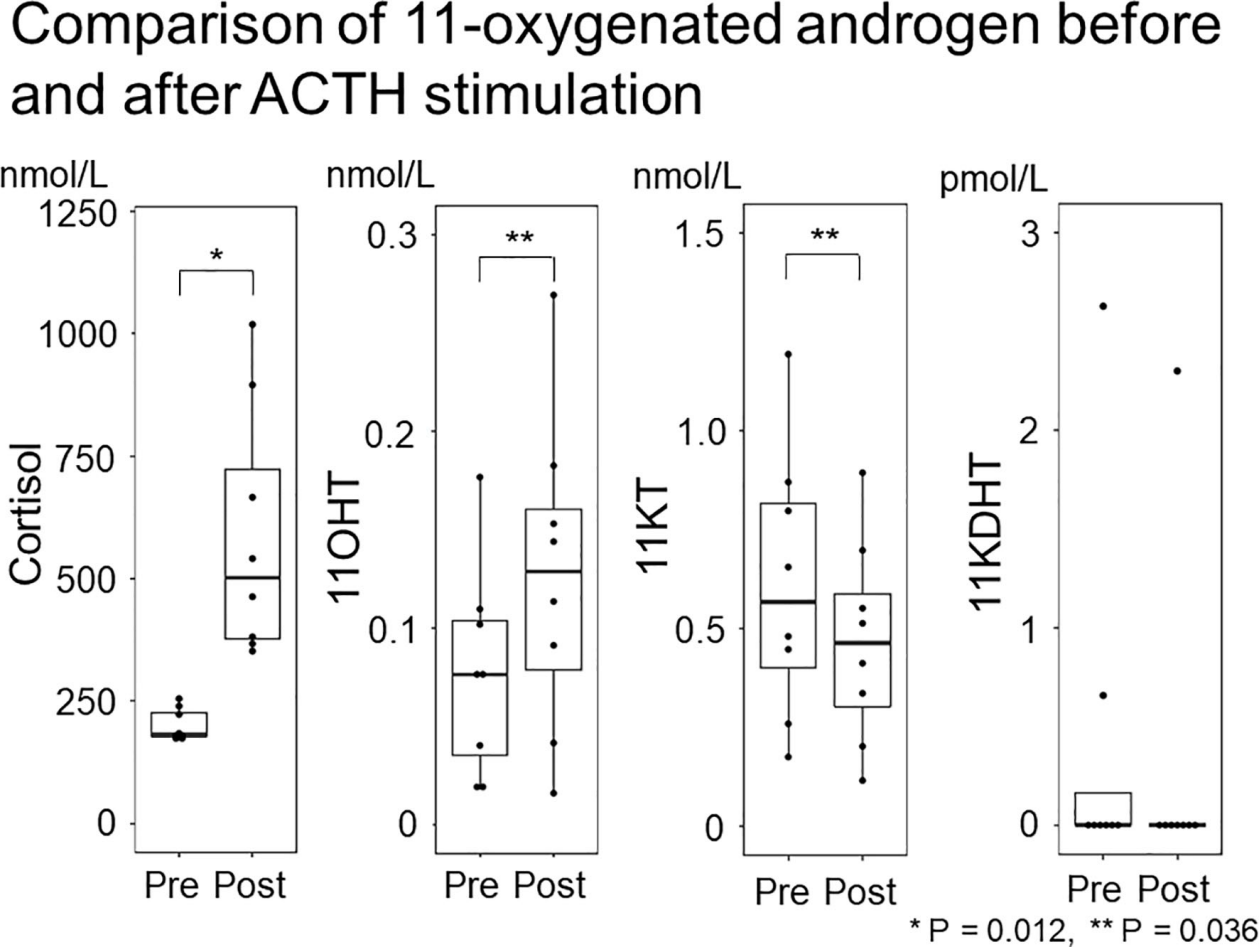
Comparison of 11-oxygenated androgen before and after ACTH stimulation. The 11-oxygenated androgen values before and after ACTH stimulation were compared. Serum cortisol and 11OHT were higher after ACTH stimulation, but 11KT did not differ before or after ACTH stimulation.

Study 3 demonstrated that 11KT did not increase after the ACTH stimulation test; indeed, the post-11KT level was significantly lower than the pre-11KT level, at 0.57 (0.31-0.85) nmol/L and 0.46 (0.23-0.66) nmol/L, respectively (**Table 5**; **Figure 5**). The 11KT level was elevated in only one patient after testing. The 11OHT level was significantly higher after the ACTH stimulation test than before it. In six of the eight patients, the 11KDHT level was lower than the assay detection limit both before and after testing.

**Table 5:** Steroid hormone values before and at 60 minutes after ACTH administration (Study 3).

	Before ACTH administration	60 minutes after ACTH administration	P value^†^
F (nmol/L), median, IQR	181.9 (175.0-235.3)	501.8 (370.4-837.8)	0.012^‡^
11KT (nmol/L), median, IQR	0.57 (0.31-0.85)	0.46 (0.23-0.66)	0.036^‡^
11OHT (nmol/L), median, IQR	0.076 (0.025-0.11)	0.13 (0.054-0.18)	0.036^‡^
11KDHT (pmol/L), median, IQR	0 (0-0.49)	0 (0-0)	0.180^‡^

ACTH, adrenocorticotropin; F, cortisol; 11KT, 11-ketotestosterone; 11OHT, 11-hydroxytestosterone; 11KDHT, 11-ketodihydrotestosterone; IQR, interquartile range.

^†^Univariate analysis was performed, and P <.05 was considered to indicate statistical significance.

^‡^Wilcoxon signed-rank test.

## 4 Discussion

The present studies examined adrenal gland involvement in 11KT production in prepubertal children. Study 1 demonstrated that the 11KT level after mPSL pulse, during which adrenal gland function was suppressed as indicated by a median cortisol value of 22.3 nmol/L, was lower than before mPSL pulse, when adrenal function was normal, suggesting that the adrenal glands play a significant role in 11KT production.

Our studies also indicated that the adrenal glands alone are insufficient for 11KT synthesis. In studies 2 and 3, the 11KT value after the CRH and ACTH stimulation tests were not elevated compared to that before the test. A previous study of adult female subjects using blood collected from the adrenal veins before and after ACTH stimulation demonstrated similar results (16). Thus, only part of the process of 11KT production occurs in the adrenal glands.

Two reactions are required to convert testosterone to 11KT synthesis, and a different enzyme catalyzes each reaction. The adrenal glands are involved in converting testosterone to 11OHT, the first process producing 11KT from testosterone. Study 1 indicated that the 11OHT value was lower after mPSL pulse than before it whereas studies 2 and 3 demonstrated higher 11OHT after CRH and ACTH stimulation. Previous studies demonstrated that CYP11B1, which converts testosterone to 11OHT, is expressed mainly in the adrenal glands (7, 10). Rege et al. also reported that 11OHT in adult females was higher after ACTH stimulation (16).

The adrenal glands are not involved to be involved in converting 11OHT to 11KT, a process known to be catalyzed by HSD11B2 (6), an enzyme not expressed in the adrenal glands (11, 12). This fact corroborated our finding that CRH and ACTH stimulation was inadequate for 11KT production. Two reasons in study 1 account for the decline in 11KT after mPSL treatment. First, 11OHT, the substrate of HSD11B2 in producing 11KT, decreased with suppression of adrenal function. Second, *HSD11B2* would have been repressed by the glucocorticoid treatment, thereby suppressing the conversion of 11OHT to 11KT (17). In studies 2 and 3, 11KT was measured only 60 minutes after CRH and ACTH stimulation; therefore, it is possible that this interval was insufficient for 11KT production in the other organs.

Our studies also suggested that the gonads are not involved in 11KT synthesis, at least in the prepubertal period. In study 1 (with prepubertal children without GnRH activation), the 11KT level was higher before mPSL pulse therapy than after it. If 11KT were exclusively produced in the gonads, its level would have been low before mPSL pulse and would not have been suppressed further after the treatment. However, our studies were unable to rule out the possibility that 11KT is synthesized in both the adrenal glands and the gonads as in a porcine model (18). Although cell culture data on gonadal 11KT production are available (2), to the best of our knowledge there are no clinical studies demonstrating 11KT production in the human gonads. In our opinion, it is unlikely that the gonads are involved in 11KT production in humans since CYP11B1 and HSD11B2 are practically unexpressed in human gonads.

Our study was not able adequately to demonstrate the involvement of the adrenal glands in the synthesis of 11KDHT, which has similar, androgenic activity to that of testosterone or 11KT (2–4). Study 2 demonstrated that the 11KDHT level was higher after than before CRH stimulation, but studies 1 and 3 failed to demonstrate any significant results. Many of the patients in studies 1 and 3 had a lower 11KDHT level than the assay detection limit; therefore, it is possible that decreases and increases in 11KDHT were unable to be assessed accurately. Two pathways produce 11KDHT (6); one converts 11OHT into 11KDHT *via* 11KT while the other does this *via* 11β-hydroxydihydrotestosterone (11OHDHT) (**Figure 1**). Since both pathways begin with 11OHT, which is produced in the adrenal glands, it is also likely that the adrenal grands are necessary in 11KDHT production.

Our studies have several limitations. First, they had a small sample size; therefore, it is possible that they had inadequate statistical power. Second, three patients with mini-puberty, when GnRH can be activated, were enrolled in study 3. However, we consider the findings of that study to be trustworthy because its results were similar to those of study 2. Third, our studies did not examine the pathway in which 11KA4 is converted from A4 *via* 11OHA4. However, a similar process may be posited for this pathway because the reactions are catalyzed by the same enzymes.

In conclusion, the adrenal glands play an important role in 11KT production in prepubertal children because 11OHT, which is converted to 11KT, is produced by the adrenal glands.

## Data availability statement

The raw data supporting the conclusions of this article will be made available by the authors, without undue reservation.

## Ethics statement

The studies involving human participants were reviewed and approved by The ethics committee at Tokyo Metropolitan Children’s Medical Center. Written informed consent from the participants’ legal guardian/next of kin was not required to participate in this study in accordance with the national legislation and the institutional requirements.

## Author contributions

KI and YH designed the study. KI collected the samples and historical data, analyzed the data, and wrote the first draft of the manuscript. YH reviewed the manuscript. All authors contributed to the article and approved the submitted version.
